# Anthropology of Infectious Disease

**DOI:** 10.3201/eid2110.151263

**Published:** 2015-10

**Authors:** Michelle Munyikwa

**Affiliations:** University of Pennsylvania, Philadelphia, Pennsylvania, USA

**Keywords:** anthrolopology, infectious disease, pathogens, bacteria, viruses, biosocial, biocultural, multispecies infections, zoonotic diseases

Merrill Singer’s Anthropology of Infectious Disease argues that pathogens are intertwined with human social worlds. Through a variety of case studies drawn from around the world, from HIV to malaria and from Lyme disease to tuberculosis, the book emphasizes a biosocial or biocultural approach to the understanding of infectious disease. In contrast to a strictly biomedical framework, the core argument of the book is that infectious diseases cannot be understood through biology alone but rather must be considered within the context of the cultural and social worlds they inhabit. The book emphasizes the interactions between biologic, political, economic, sociocultural, and ecologic factors and how these factors affect the emergence, prevention, treatment, distribution, cultural experiences, and global impact of pathogens.

The first 2 chapters of the book provide the framework for the rest of the text. The first chapter begins with a history of the anthropology of infectious disease, starting from the post–World War II period to the present day. This chapter continues by making a strong case for the inclusion of anthropologic frameworks in our understanding of microbes and their impact because ethnography and other anthropologic tools link every day on-the-ground experiences to broader social structures and global processes. Following this foundation in social science, the book’s second chapter provides a more biologically based introduction to microbes, emphasizing human–pathogen interaction and evolution.

Using theoretical approaches ranging from ecosocial theory, medical ecologic theory, phenomenologic and meaning-centered approaches, and critical medical anthropology, Singer explores a wide array of topics throughout the rest of the book. Drawing from medical ecologic theory, the next 3 chapters explore connections between humans, the environment, and other organisms. These linkages are explored through the lens of multispecies infections and zoonotic diseases, the effect of environmental devastation on patterns of disease, and the evolutionary arms race between humans and emerging drug-resistant pathogens. Moving on to the role of social factors in the differential burden of disease in various populations, the last sections of the book explore social experiences of suffering, highlighting the interaction of infectious with noninfectious disease, the relationship between inequality and emerging infectious disease, and the effects of politics and global structural forces on future pathogenic challenges.

Throughout the text, Singer presents a broad array of examples. The volume of case studies drawn upon, although useful in their analytic breadth, could be overwhelming for the novice reader. Thus, the book could benefit from an emphasis on a few core case studies.

Overall, however, Anthropology of Infectious Disease is written clearly and compellingly and would make an important addition to a course in public health, medical anthropology, or even microbiology. Although the book could be assigned as a whole, each chapter can stand alone, making it a versatile and practical teaching tool for students at multiple levels. In addition, the book contains a comprehensive glossary and discussion questions at the end of each chapter, making it even more useful in educational settings. Because of its accessible style and clear elucidation of theory, this book is also appropriate for practitioners in medicine or public health and infectious disease who would like to familiarize themselves with social science approaches in the field.

